# Genetic analysis of oligo-recurrence breast cancer: correlation with clinical outcomes

**DOI:** 10.1186/s12885-023-10833-2

**Published:** 2023-09-15

**Authors:** Kuikui Jiang, Danyang Zhou, Fei Xu, Wen Xia, Qiufan Zheng, Qianyi Lu, Rongzhen Luo, Ruoxi Hong, Shusen Wang

**Affiliations:** 1 Department of Medical Oncology, State Key Laboratory of Oncology in South China, Collaborative Innovation Center for Cancer Medicine, Sun Yat-sen University Cancer Center, Guangzhou, 510060 China; 2 Department of Oncology, Peking University Shenzhen Hospital, Shenzhen, China; 3 Department of Pathology, State Key Laboratory of Oncology in South China, Collaborative Innovation Center for Cancer Medicine, Sun Yat-sen University Cancer Center, Guangzhou, 510060 China

**Keywords:** Oligo-metastatic disease, Oligo-recurrence, Breast cancer, Genetic analysis

## Abstract

**Background:**

We aimed to identify the relationship between the genomic characteristics and clinical outcomes of oligo-metastatic breast cancer.

**Methods:**

Oligo-metastatic breast cancer diagnosed by pathology from January 2001 and August 2019 were reviewed and we matched the poly-metastatic patients based on the clinicopathological features of patients included. Clinicopathological values and data of genomic alterations were collected. Oligo-recurrence (oligo-R) was defined as a situation where disease progression occurred in less than 5 anatomical sites and other anatomic areas still suppressed by the ongoing therapy.

**Results:**

A total of 26 breast cancer patients were enrolled in our study, including 14 patients with strict oligo-metastatic disease (oligo-R > 6 months) and 12 with simultaneous poly-metastatic disease. PIK3CA, TP53 and ERBB2 were the most common shared alterations identified in patients included. Based on the median time of oligo-R, we divided the patients with oligo-metastasis into longer oligo-R group (oligo-R > 31.04 months) and shorter oligo-R group (oligo-R ≤ 31.04 months). The analysis of PIK3CA mutation sites showed that H1047R mutation was closely associated with oligo-metastasis, rather than poly-metastasis. H1047R mutation also predicted a better prognosis (oligo-R > 31.04 months) in oligo-metastatic breast cancer. In addition, HER2 positive was more likely to be related to a good outcome in patients with oligo-metastasis.

**Conclusions:**

Through the genetic analysis of samples from oligo-metastasis, we found the prognostic values of PIK3CA H1047R and HER2 in oligo- and poly-metastasis. We improved the stratification of prognosis and provided new insights for biological behaviors of oligo-metastatic breast cancer.

## Background

Breast cancer is the most common malignancy in women. The incidence rate and mortality rate account for 24.5% and 15.5% respectively, both ranking the first in female cancers in 2020 worldwide [1]. According to the expression of hormone receptor (HR), human epidermal growth factor receptor 2 (HER2) and Ki-67, breast cancer can be divided into different subtypes with different treatment strategies and survival in clinical practice. Despite improvement in early detection and treatments, approximately 30% of breast cancer patients will finally develop metastatic disease [2] and metastatic breast cancer (MBC) remains the dismal prognosis with a median overall survival (OS) of ~ 3 year and a 5-year survival of only ~ 25% [3, 4].

Oligo-metastatic breast cancer is a special condition of MBC. Approximately 1 ~ 10% of newly diagnosed MBC patients experience this oligo-metastatic disease [5, 6]. In the 4th ESO-ESMO International Consensus Guidelines for Advanced Breast Cancer (ABC4), oligo-metastatic disease is defined as low volume metastatic disease with limited number and size of metastatic lesions (up to five and not necessarily in the same organ) [7]. Notably, oligo-metastatic disease is potentially amenable for local treatment, aimed at achieving a complete remission status and a potential long-term survival [8–10]. With the further understanding of oligo-metastatic disease, the idea of oligo-recurrence (oligo-R) has been proposed [11]. According to previous studies, oligo-recurrence has become an internationally agreed term, which is referred to cancer patients have 1 to 5 metastatic or recurrent lesions that could be treated by local therapy, with controlled primary lesions [12–17].

Breast cancer is a heterogeneous disease [18, 19]. Although the overall prognosis of patients with oligo-metastasis is better than that of patients with poly-metastasis, there are still some patients with poor prognosis. Aberrant mutations are commonly identified in patients with breast cancer, especially MBC. Screening for genomic mutations and alterations may identify patients with different disease progression and prognosis. High-throughput sequencing, commonly known as next-generation sequencing (NGS) is now readily available for clinical use [20, 21] due to the improvement of reliability and affordability of NGS after the success of The Cancer Genome Atlas (TCGA) Project. For breast cancer patients with oligo-metastatic disease, it is necessary to describe the genomic characteristics in order to identify patients in different level of risk and individualize clinical prognosis and treatment decisions. Based on the above, we performed the genetic analysis of oligo-metastatic and poly-metastatic patients, aiming to identify the relationship between the genomic characteristics and clinical outcomes of oligo-metastatic breast cancer and provide suggestions to the management of oligo-metastatic breast cancer.

## Methods

### Patient population

Patients with breast cancer at Sun Yat-sen University Cancer Center between January 2001 and August 2019 were retrospectively reviewed. Only patients meeting all of the following criteria were included: (1) breast cancer patients with histologically confirmed diagnosis, (2) patients with oligo-metastatic disease, (3) patients with sufficient pathological tissue to perform NGS (FoundationOne CDx). Patients with any malignancies besides breast cancer were excluded. Then, we matched the patients with poly-metastases in the same period according to the clinicopathological features of the patients with oligo-metastases included. For each patient, clinicopathological data (age, gender, pathology, TNM stage, metastatic sites and treatment strategies) were collected and the results of NGS (genomic findings, microsatellite status (MS), tumor mutational burden (TMB) and variants of unknown significance (VUS)) were analyzed. Oligo-recurrence was defined as a situation in which disease progression occurred in less than 5 anatomical sites and other anatomic areas still suppressed by the ongoing therapy, or last follow-up (censored). All patients included were followed-up until death or study data cutoff (March 2020). The study was approved by the Ethical Committees of Sun Yat-sen University Cancer Center (NO.: B2020-145-01) and individual consent for this retrospective analysis was waived. All methods were carried out in accordance with relevant guidelines and regulations.

### Tumor tissue analysis

Pathological specimens were reviewed by the experienced pathologist. Specimens were stained for estrogen receptor (ER) and progesterone receptor (PR) by immunohistochemistry (IHC) and HER2 by IHC and fluorescence in situ hybridization (FISH) according to current guideline (available at www.nccn.org/). Specimens then underwent FoundationOne CDx. In brief, DNA was extracted from formalin-fixed, paraffin-embedded (FFPE) tumor samples, 50-1000 ng of which underwent whole-genome shotgun library construction, and detection of alterations in a total of 324 genes was included. Assay specifications were determined for typical median exon coverage of approximately 500X. Sequence data were analyzed through a computational analysis pipeline to accurately detect all classes of genomic alterations, including substitutions, indels/deletions, copy number amplifications and selected genomic rearrangements.

### Statistical analysis

Clinicopathological variables and characteristics of NGS of patients were summarized using descriptive statistics, the latter was from a database of all genomic alterations based on the FoundationOne CDx reports. Differences between categorical variables were determined using the Chi-square test. Survival analyses were calculated by the Kaplan–Meier method. Statistical analysis was performed using SPSS version 25.0. All *P* values were two-sided, and *P* values < 0.05 were considered significant for all statistical analyses.

## Results

### Characteristics of patients

A total of 26 MBC patients were included in our study between January 2001 and August 2019. Among them, 14 breast cancer patients were oligo-metastatic (The length of oligo-R was longer than 6 months in all patients, suggesting the patients included is a relatively strict oligo-metastatic status rather than a pre stage of poly-metastasis.) and 12 patients were simultaneous poly-metastatic. Clinicopathologic and genomic characteristics of patients were presented in Table 1. All patients included were female and there was no significant bias in clinical factors such as age, subtype and TNM stage between oligo- and poly-metastasis groups. No significant difference was showed in number of gene alteration, TMB and VUS, actional mutation in two groups. The MS of all patients was stable. The median number of treatment lines for 12 patients with poly- metastatic breast cancer was 3. All patients with oligometastatic breast cancer had their primary lesions treated with radical surgery. Fourteen patients included received postoperative adjuvant chemotherapy based on anthracycline, cyclophosphamide and paclitaxel, and 10 of them conducted adjuvant radiotherapy. For oligometastatic lesions, 12 patients received local treatment, including 11 patients who performed surgical resection, 2 of them who received radiotherapy for oligometastatic lesions, and 1 patient who conducted interventional treatment of liver. Further analysis was performed in patients with oligo-metastatic disease shown in Table 2. Lung was the most common site of metastasis in patients included and the median time of oligo-R of patients included was 31.04 months (range: 7.1–84.2 months).

**Table 1:** Clinicopathological and genomic characteristics of patients

Factor		Total N = 26	Oligo-metastasis N = 14	Poly-metastasis N = 12	*P*
Age	Median (Range)	42 (31–67)	40 (31–65)	46 (36–67)	*
Subtype	HR + HER2-	15	7	8	0.310
	HER2+	8	6	2	
	TNBC	3	1	2	
T	≤ 2	15	8	7	1.000
	> 2	11	6	5	
N	≤ 1	13	7	6	1.000
	> 1	13	7	6	
Specimen site	Primary tumor	16	8	8	0.701
	Metastatic sites	10	6	4	
Gene alterations	Median (Range)	4.5 (2–13)	4.5 (2–7)	5 (2–13)	*
TMB	Low	20	11	9	1.000
	Intermediate	6	3	3	
VUS	Median (Range)	9.5 (4–20)	11 (4–15)	9 (6–20)	*
Actionable mutation	Yes	22	13	9	0.306
	No	4	1	3	

Abbreviation: HR, hormone receptor; HER2, human epidermal growth factor receptor 2; TNBC, Triple negative breast cancer; T, Tumor; N, Node; TMB, Tumor mutational burden; VUS, Variants of unknown significance

* means Chi-square test was not carried out

**Table 2:** Characteristics of patients with oligo-metastatic disease

Factor		Oligo-metastasis N = 14
Metastatic sites	Lung	10
	Liver	3
	Chest wall	1
Oligo-recurrence	Median (Range)	31.04 (7.1–84.2)
Therapy given	CT + TT	2
	CT + LT	9
	CT + TT + LT	3

Abbreviation: CT, Conventional therapy (including conventional chemotherapy and endocrine therapy); TT, Targeted therapy; LT, Local treatment (including surgical resection, radiotherapy and interventional treatment)

### Genomic analyses of patients

**Fig. 1:**
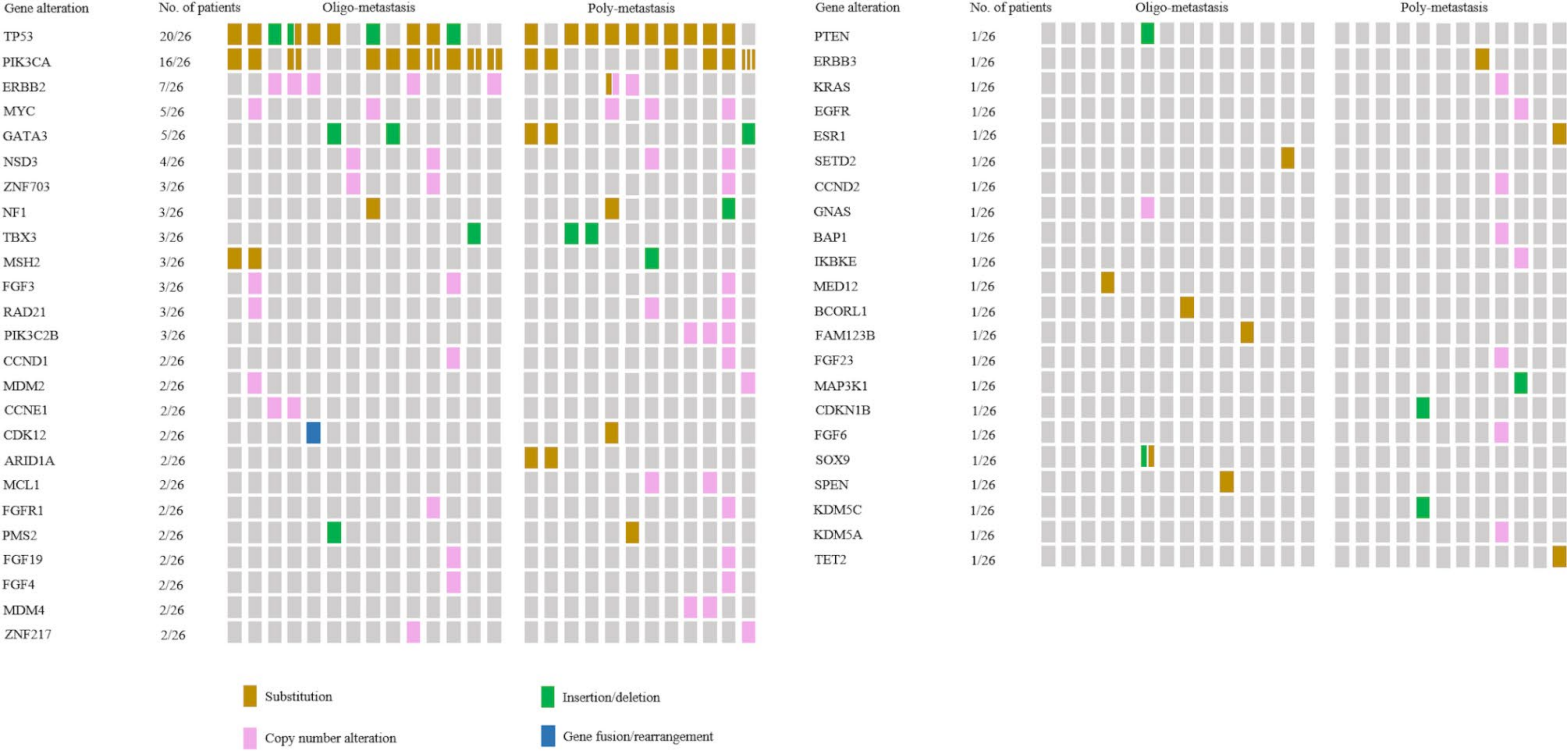
Oncoprint of somatic gene alterations in biopsies of 26 breast cancers Shown are the distribution of gene alteration identified by NGS in the 26 lesions from breast cancer patients. Alterations include point mutations and copy number alterations as shown in the key below

**Fig. 2:**
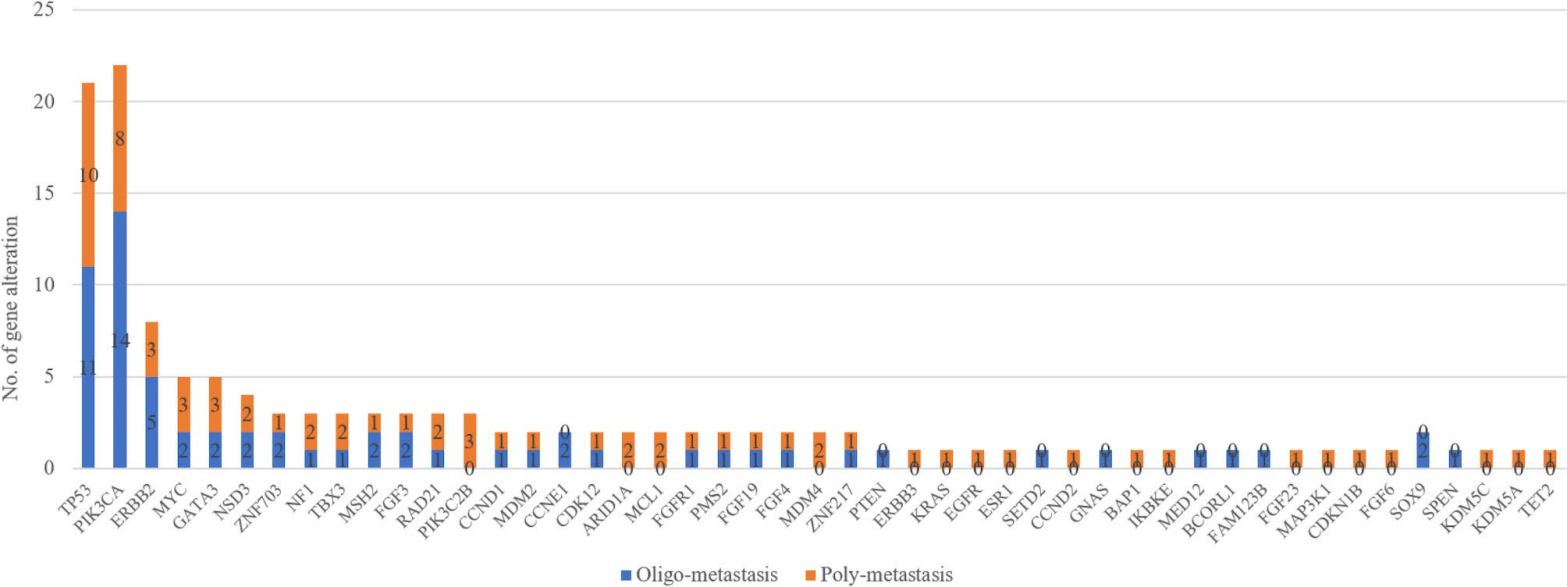
Number of gene alteration based on gene type classified by disease status

The overall genomic distribution of patients was showed in Fig. 1. The sum of gene alteration in oligo-metastasis and poly-metastasis was 64 and 69 respectively, and the median values of gene alteration was 4.5 and 5 respectively. The most common shared alterations identified were PIK3CA, TP53 and ERBB2 observed in Fig. 2: PIK3CA mutations (n = 22, oligo-metastasis vs. poly-metastasis = 14 vs. 8), TP53 mutations (n = 21, oligo-metastasis vs. poly-metastasis = 11 vs. 10) and ERBB2 mutation or amplification (n = 8, oligo-metastasis vs. poly-metastasis = 5 vs. 3). According to the class of genomic alterations, the number of substitution, insertion/deletion, copy number alteration and gene fusion/rearrangement were 30 vs. 29, 10 vs. 8, 23 vs. 32 and 1 vs. 0 in oligo- and poly-metastasis, respectively.

### PIK3CA and prognosis

**Fig. 3:**
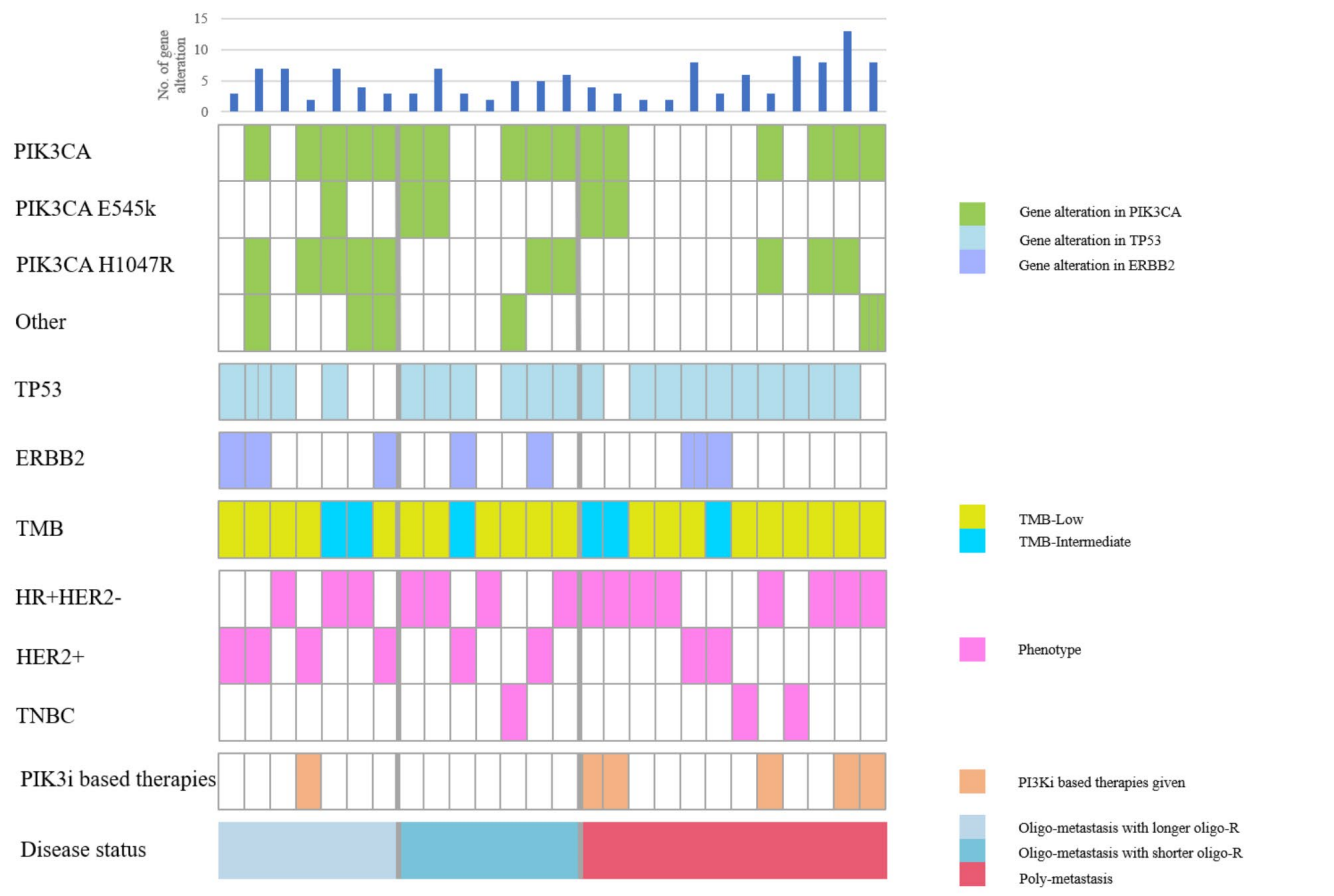
Clinicopathologic and genomic characteristics of patients associated with PIK3CA mutation

**Fig. 4:**
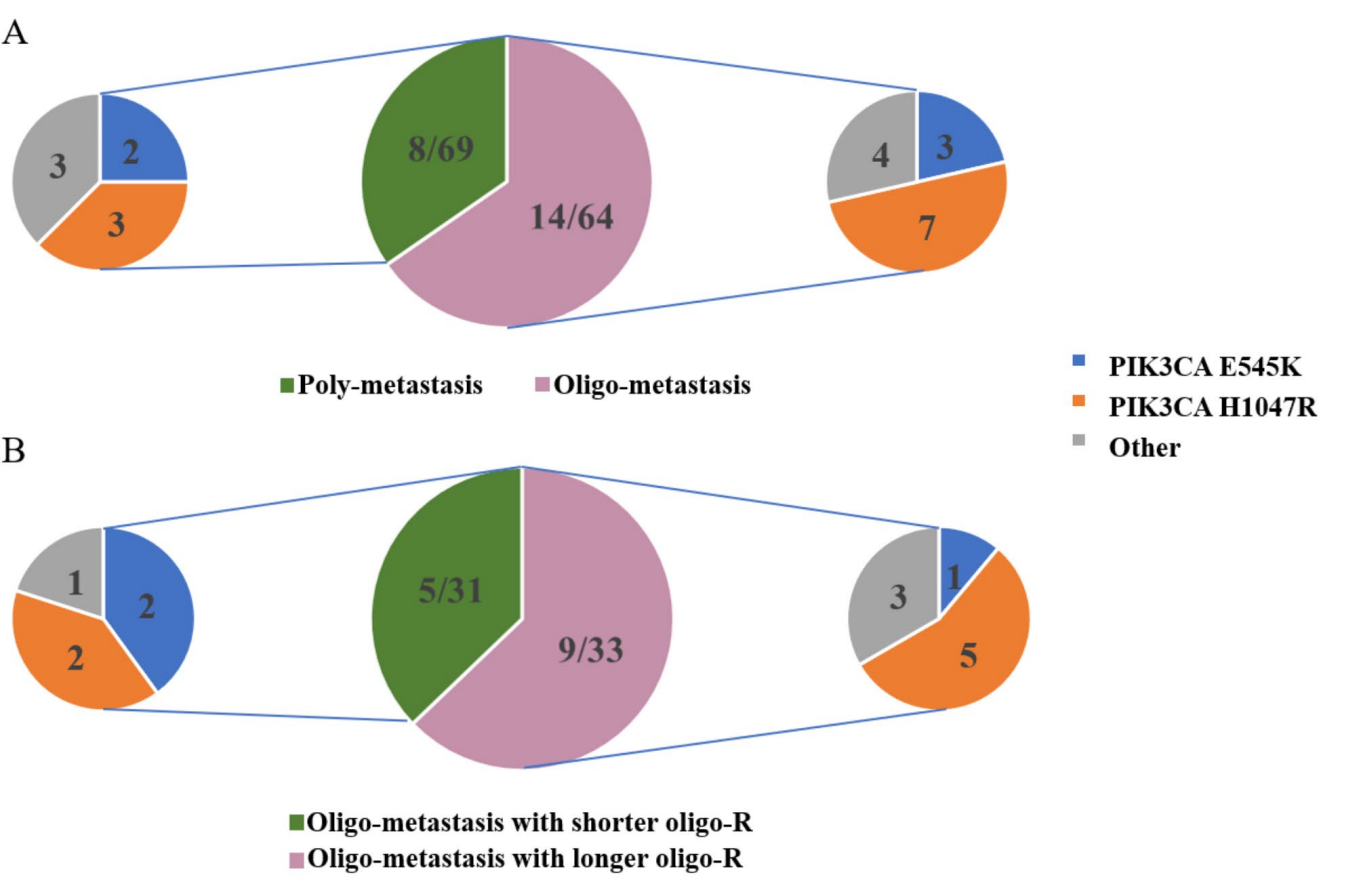
The distribution of PIK3CA mutations in patients with metastatic breast cancer. (**A**) The distribution of PIK3CA mutations in oligo- and poly-metastasis. (**B**) The distribution of PIK3CA mutations in longer oligo-R group and shorter oligo-R group

Due to the high incidence of PIK3CA gene alterations and mutations involving PIK3CA mainly concentrated in H1047R and E545K, analysis of prognostic values of PIK3CA was carried out. Clinicopathologic and genomic characteristics of patients were presented in Fig. 3. The analysis of H1047R and E545K suggested that PIK3CA H1047R was the main mutation site in oligo-metastasis, accounting for 50% (7/14) in PIK3CA mutation, compared to poly-metastasis (37.5%, 3/8) (*P* = 0.675). The number of PIK3CA E545K in oligo-metastasis and poly-metastasis was not significantly different, 21.4% (3/14) and 25.0% (2/8, *P* = 1.000), respectively (Fig. 4A). Based on the median value of oligo-R, we divided the patients with oligo-metastasis into longer oligo-R group (oligo-R > 31.04 months) and shorter oligo-R group (oligo-R ≤ 31.04 months). The sum of gene alteration in longer oligo-R group and shorter oligo-R group was 33 and 31, respectively. Similarly, PIK3CA gene alteration is also more common in patients with longer oligo-R (9/33) than that in patients with shorter oligo-R (5/31). PIK3CA H1047R is more common in patients with longer oligo-R (5/9) than that in patients with shorter oligo-R (2/5, *P* = 1.000). The number of PIK3CA E545K in shorter oligo-R (2/5) was more than that in longer oligo-R (1/9, *P* = 1.525, Fig. 4B).

### ERBB2/HER2 and prognosis of oligo-metastasis

**Fig. 5:**
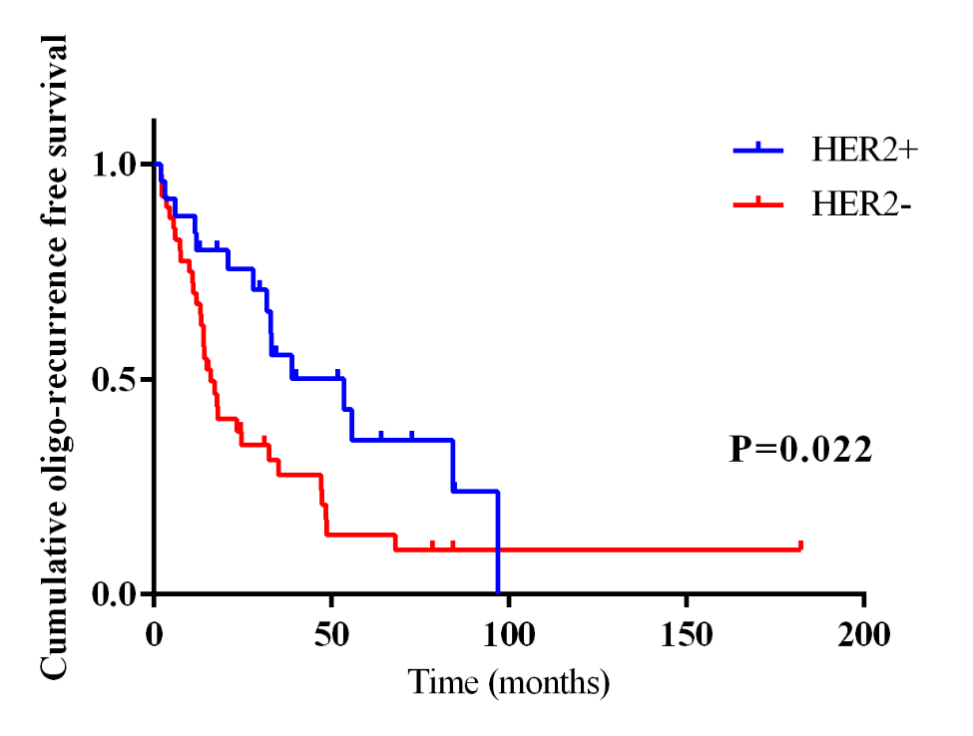
Kaplan–Meier curve for oligo-R of breast cancer patients stratified by HER2.

ERBB2 alterations were found in 3 patients in longer oligo-R group and 2 in shorter oligo-R group. There were 4 HER2 positive patients and 2 patients in longer and shorter oligo-R group, respectively. One patient with HER2 positive breast cancer in longer oligo-R group was performed FoundationOne CDx using the specimen taken from metastasis site, and no ERBB2 mutation was found. HER2 positive seemed to be more common in longer oligo-R group. HER2 status determined by NGS showed 97% accuracy relative to the HER2 status measured by FISH [22]. Based on this, we expanded the size of sample to explore the relationship between HER2 and the prognosis of patients with oligo-metastasis. The expanded data came from our previous study on liver oligo-metastasis in breast cancer [23] and we extracted HER2 positive (25 cases) and HER2 negative case (40 cases) based on the primary site by using IHC and FISH for subsequent analysis. Among patients with HER2 positive breast cancer, 80% of patients treated with anti-HER2 therapy. Kaplan-Meier analysis suggested that HER2 positive patients had a longer oligo-R, compared to the HER2 negative disease (*P* = 0.022, Fig. 5).

### Variants of unknown significance and prognosis

As the clinical significance of VUS needs to be further explored, we analyzed the distribution of VUS in patients included. The sum of VUS in longer oligo-R group, shorter oligo-R group and poly-metastasis was 69, 76 and 121 respectively. All of the 7 VUSs related to ARID1A were detected in poly-metastasis. Similarly, 3 VUSs involving WT1 were all found in poly-metastasis, not oligo-metastasis. VUSs on MTOR and IGF1R only occurred in patients with longer oligo-R.

## Discussion

While there have been extensive studies into the molecular characteristics of MBC, little is known regarding genomic alterations of oligo-metastatic disease and their relation to clinical outcomes. At present, the investigations on oligo-metastasis genes are mainly focused on liver oligo-metastatic colorectal cancer (CRC). TP53 and KRAS mutation are related to a high risk of local failure and poor survival [24, 25]. Unfortunately, there is a lack of clear and uniform gene marker for oligo-metastatic disease of other tumors, including breast cancer. In our study, PIK3CA H1047R mutation was associated with oligo-metastatic disease, not poly-metastatic disease. And this mutation also predicted a good prognosis in patients with oligo-metastatic breast cancer. HER2 positive patients with oligo-metastasis was more likely to have a good prognosis, compared patients with HER2 negative. In addition, VUS might also be a potential prognostic biomarker in metastatic disease.

According to TCGA, PIK3CA (coding mutations in 40.1% of the samples) dominated the mutation landscape of breast cancer [21]. PIK3CA gene, which is located on chromosome 3 (3q26.32), encode the α isoform of catalytic subunit phosphatidylinositol-4, 5-bisphosphate 3-kinase (PI3K). The mutations in helical and kinase domains lead to increased PI3K activity and the activity of PI3K has downstream effects on the AKT and mTOR pathways that control cell cycle and metabolism in cancer progression [26]. Despite the pre-clinical evidence that PIK3CA is an oncogene, results on the relation between the PIK3CA mutation and outcomes are inconsistent in clinical studies, demonstrating worse outcomes in breast cancer patients with PIK3CA mutations, no differences in outcomes by mutation status and better outcomes for HR positive breast cancer with PIK3CA mutations recently [27–29]. In our study, PIK3CA mutation was more in oligo-metastasis than in poly-metastasis and was also more in oligo-metastasis with longer oligo-R than in shorter oligo-R, suggesting PIK3CA mutation may be related to good prognosis in oligo-metastatic disease. There is one possible reason that alterations in different exons of PIK3CA have varying impacts on tumor development and progression and differ in prognostic value [30–32]. Within PIK3CA, H1047R (the kinase domain) and E545K (the helical domain) are most common hotspot mutations. Compared with E545K, which relies on Ras-GTP rather than p85, H1047R is highly dependent on p85 for its oncogenic capacity but independent of Ras-GTP [33]. Arman et al. found that E545K markedly promoted proliferation, survival, cytoskeletal reorganization, migration, and spheroid formation, whereas H1047R only enhanced the first three [34]. PIK3CA E545K mutations, but not PIK3CA H1047R mutations, preferentially activate AKT1 signal [35]. In addition, previous clinical analysis also suggests that PIK3CA E545K is independently associated with early recurrence and death, whereas PIK3CA H1047R is associated with optimal prognosis in infiltrating lobular carcinomas [30]. PIK3CA H1047R mutants are strongly associated with lymph-node negativity [31], which contributes to good prognosis in some degree. That corresponds to our results. In our research, the distribution of PIK3CA H1047R mutation suggested this mutation might be related to good prognosis in oligo-metastatic disease, whether in oligo-metastasis and poly-metastasis groups, or in oligo-metastasis with longer oligo-R and oligo-metastasis with shorter oligo-R. Notably, although chemotherapy before sample collection may have an impact on PIK3CA mutations, the PIK3CA mutations detected were more evenly distributed in primary lesions vs. metastatic lesions and pre-treatment vs. post-treatment, suggested that treatments have little effect on analysis of PIK3CA mutation in our study.

Although HER2 positive breast cancer is associated with aggressive progression, it is now increasingly apparent that HER2 positive breast cancer is clinically and biologically heterogeneous [36–39]. Great variability of patient’s response and survival outcomes following anti-HER2 therapy [40, 41] and high biological variability [42] are common. Clinical HER2 positive breast cancer is divided into different intrinsic subtype based on molecular data derived from DNA, RNA and protein. Although clinical HER2 positivity measured by IHC and FISH is mainly determined as the HER2-enriched subtype, all of the intrinsic subtypes can be identified within clinical HER2 positive breast cancer [21, 37, 38]. In addition, intratumoral heterogeneity of HER2 gene amplification can contribute to inaccurate assessment of HER2 status and increase the inconsistency of clinical response [43, 44]. On the other hand, the prognostic landscape for HER2 positive BC patients has considerably improved due to the advent of anti-HER2 therapies. HER2 antibodies and their derivatives such as trastuzumab [45, 46], pertuzumab [47] and trastuzumab-emtansine (T-DM1) [48], as well as the tyrosine kinase inhibitors (TKIs) such as lapatinib [49, 50] and pyrotinib [51], have become the standard treatments for metastatic HER2 positive breast cancer. In this study, most patients received anti-HER2 therapy and a few patients used more than one anti-HER2 drugs, which prolonged the progression of disease to some extent. That may partly explain why HER2 positive patients with oligo-metastasis was more likely to have a good prognosis. Previous reports showed that 27% of patients with HER2 positive, locally advanced or metastatic breast cancer who commenced first line trastuzumab-containing therapy may be long-term responders (beyond 2 years) [52], and nearly half of the patients remained in remission for more than 5 years in patients who had non-progressive disease for at least 2 years on trastuzumab [52, 53].

Significant numbers of variants labeled only as VUSs are detected in cancer patients [54, 55]. There is not enough information to classify the VUSs as definitively pathogenic or benign due to the rarity of the finding and the insufficient epidemiological evidence at the time of the test [56]. This ambiguity leads to the significant diversity in management for patients with VUSs [57]. In order to explore the clinical values, we tried to analyze the relationship between VUS and prognosis of patients with oligo-metastasis, and found that there was a trend between VUSs related to some genes and specific prognosis of oligo-metastatic disease. Although it would be inappropriate to accept these recurrent variants as pathogenic or benign, they may warrant higher priority than other observed VUS’s.

Our study is limited by small size of sample and retrospective approach. Retrospective analysis may have missing or erroneous data entry. Some subgroups analyzed may have insufficient sample size to identify significant differences due to small sample size. Therefore, further large-scale multicenter prospective studies are needed to confirm our findings. In addition, some samples measured by the FoundationOne CDx were taken from primary sites when the disease did not develop metastasis. The progress of the disease might lead to changes in gene expression, thus reducing the persuasiveness of the results. Notably, we matched the breast cancer patients with oligo-metastasis and poly-metastasis and compared the differences in the genomic characteristics in the present study. Further, we also analyzed the genomic characteristics of oligo-metastatic patients with different prognosis. On the other hand, the oligo-recurrence of the oligo-metastasis patients we included is relatively long, suggesting the patients included is a relatively strict oligo-metastatic status rather than a pre stage of poly-metastasis. The current research will help to reveal the difference in genomic characteristics between poly-metastatic breast cancer and oligometastatic breast cancer, and the relationship between internal heterogeneity and gene expression in oligometastatic breast cancer, providing the reference for further mechanism exploration.

## Conclusions

Increasing attention has been paid to oligo-metastatic breast cancer due to the potential curability and the unclear mechanism. The development of high-throughput sequencing technology also enables us to perform genetic analysis on oligo-metastatic disease quickly and accurately. Through the genetic analysis of samples from oligo-metastasis, we found the prognostic values of PIK3CA H1047R, HER2 and VUS in oligo-metastasis, as well as common shared alterations in oligo- and poly-metastasis. In order to further verify and clarify the biological basis, more mechanism studies and large-scale translational researches are needed.

## Data Availability

The data generated and analysed during the current study are available in the Sequence Read Archive repository, accession number: PRJNA743504.
